# Metabolome and transcriptome analyses reveal changes of rapeseed in response to ABA signal during early seedling development

**DOI:** 10.1186/s12870-024-04918-8

**Published:** 2024-04-05

**Authors:** Yaqian Chen, Jinfeng Wu, Changrui Ma, Dawei Zhang, Dinggang Zhou, Jihong Zhang, Mingli Yan

**Affiliations:** 1 School of Life and Health Sciences, Hunan University of Science and Technology, Xiangtan, 411201 China; 2 Yuelushan Laboratory, Changsha, 410125 China; 3 Hunan Research Center of Heterosis Utilization in Rapeseed, Crop Research Institute, Hunan Academy of Agricultural Sciences, Changsha, 410125 China; 4 Hunan Key Laboratory of Economic Crops Genetic Improvement and Integrated Utilization, Hunan University of Science and Technology, Xiangtan, 411201 China

**Keywords:** Rapeseed, Abscisic acid, Seed germination, Metabolomics, Transcriptome

## Abstract

Seed germination is an important development process in plant growth. The phytohormone abscisic acid (ABA) plays a critical role during seed germination. However, the mechanism of rapeseed in response to ABA is still elusive. In order to understand changes of rapeseed under exogenous ABA treatment, we explored differentially expressed metabolites (DEMs) and the differentially expressed genes (DEGs) between mock- and ABA-treated seedlings. A widely targeted LC-MS/MS based metabolomics were used to identify and quantify metabolic changes in response to ABA during seed germination, and a total of 186 significantly DEMs were identified. There are many compounds which are involved in ABA stimuli, especially some specific ABA transportation-related metabolites such as starches and lipids were screened out. Meanwhile, a total of 4440 significantly DEGs were identified by transcriptomic analyses. There was a significant enrichment of DEGs related to phenylpropanoid and cell wall organization. It suggests that exogenous ABA mainly affects seed germination by regulating cell wall loosening. Finally, the correlation analysis of the key DEMs and DEGs indicates that many DEGs play a direct or indirect regulatory role in DEMs metabolism. The integrative analysis between DEGs and DEMs suggests that the starch and sucrose pathways were the key pathway in ABA responses. The two metabolites from starch and sucrose pathways, levan and cellobiose, both were found significantly down-regulated in ABA-treated seedlings. These comprehensive metabolic and transcript analyses provide useful information for the subsequent post-transcriptional modification and post germination growth of rapeseed in response to ABA signals and stresses.

**Supplementary Information:**

The online version contains supplementary material available at 10.1186/s12870-024-04918-8.

## Introduction

Rapeseed (*Brassica napus L.*) is used for the production of food, bio-fuels and industrial compounds [1, 2]. It occupies position of being the most extensively cultivated oilseed crop in global. According to the Triangle of U3, *Brassica napus* (2n = 4x = 38, AACC) is an allopolyploid crop that inherits two sets of chromosomes (AA) from *Brassica rapa* and two sets (CC) from *Brassica oleracea* with the proceed of interspecific hybridization [3]. A series of genetic studies have been conducted on the evolution and process of rapeseed [4, 5]. In recent years, there have been many studies exploring the physiological characteristics, gene regulatory networks [6], and breeding methods of rapeseed [7].

Abscisic acid, also known as ABA, is a phytohormone that plays a crucial role in regulating various physiological processes in plants. It has been found to be involved in processes such as seed development and dormancy, stomatal closure, and stress response [8, 9]. It is triggered by environmental stresses, and it helps plants adapt and survive in unfavorable conditions. Understanding the functions of ABA can provide valuable insights into plant physiology and the strategies for crop improvement and stress tolerance [10]. During seed development, ABA induces embryonic growth arrest during the transition period from embryogenesis to reserve accumulation, and then induces primary dormancy, thereby preventing survival and allowing seeds to spread in a dormant state [11]. Seed germination plays an critical role in crop production, and includes a series of complex biochemical and molecular processes [12]. The hormone balance of abscisic acid (ABA) and gibberellic acid (GA) plays a key role in many physiological processes during seed germination. High ABA: GA ratio will maintain dormancy [13]. Many studies have shown that the antagonism of Abscisic acid (ABA) and gibberellin (GA) is of great significance for seed dormancy and germination, the dormant seed state is induced and maintained by ABA and released by GA [14]. Studies have shown that ABA acts as a germination inhibitor and GA acts as a germination promoting hormone in several species [15]. From a biomechanical perspective, the key part of seed germination is the interaction between controlling embryonic growth and surrounding mechanical forces that inhibit endosperm tissue [16]. It is generally believed that weakened endosperm is a prerequisite for the emergence of embryonic roots [17, 18]. The antagonistic effects of GA and ABA are also applicable to endosperm rupture. It has been shown that endosperm rupture is promoted by GA and inhibited by ABA [14].

Metabolome can quantitatively measure the dynamic multiparameter response of the living system to pathophysiological stimuli or genetic modification, thereby gaining new insights into biological processes such as aging, disease onset and progression [19]. It has been widely used in plants with complex genome, such as apple [20] to identify metabolites and obtain their information. On the basis that transcriptome sequencing technology has been widely used for the detection of genes and markers in many plants [21]. There have been studies on the correlation between differentially expressed metabolites (DEMs) and differentially expressed genes (DEGs) using the association analysis method of transcriptome and metabolome to further understand the mechanisms of plant development.

ABA plays a prominent role in inhibiting seed germination. However, there have been no profound reports on the potential molecular mechanisms of ABA induced seed stress response and further inhibition of seed germination in rapeseed. In this study, we aimed to obtain differential metabolites in response to ABA stress in rapeseed. In addition, the correlation between DEMs and DEGs in the process of exogenous ABA inhibiting seed germination was studied through the combination analysis of metabolomics and transcriptome. We further investigate more significant metabolic pathways which are involved in ABA-mediated early seedling development. The association analyses based on metabolome and transcriptome provides useful information for the subsequent post-transcriptional modification and post germination growth of rapeseed in response to ABA stimuli.

## Materials and methods

### Plant material and germination conditions

The rapeseed cultivar of zhongshuang 11 (hereafter ‘ZS11’) is a commercial elite inbred line. The Oil Crops Research Institute, Chinese Academy of Agricultural Science provides this inbred line to many laboratories including our lab (School of Life and Health Sciences, Hunan University of Science and Technology). ZS11 has been sequenced by Huazhong agricultural university, Hubei Province, China [22]. In this study, the accession of ZS11 with uniform size and no obvious defects were used as experimental materials. Three independent biological replicates were performed for each treatment. For each biological replications, 50 seeds were incubated in a petri dish (35 mm) with 1.5 mL ddH_2_O. After 2 days cultivation, the germinated seeds were sprayed with ddH_2_O or100 µM Abscisic acid (ABA). All of the seedlings used in the experiments were grown at 24°C under 16/8 hours light/dark conditions.

### LC-MS/MS analysis

The samples were harvested after 3 h mock and ABA treatment. All of mock and ABA treatment groups for metabolome contained 6 biological replicates. Samples and grinding beads are added to a centrifuge tube to obtain the extraction solution. The LC-MS/MS analysis was performed by Majorbio Bio-Pharm Technology Co. Ltd. (Shanghai, China) [23].

### Metabolomics analyses

The data matrix obtained by searching database was uploaded to the Majorbio cloud platform (https://cloud.majorbio.com) for data analysis. The principal component analysis (PCA) and orthogonal least partial squares discriminant analysis (OPLS-DA) were performed. The metabolites with Variable importance in the projection (VIP) > 1, *p* < 0.05 were determined as significantly different metabolites based on the VIP obtained by the OPLS-DA model and the p-value generated by student’s t test [24]. Differential metabolites among two groups were mapped into their biochemical pathways through metabolic enrichment and pathway analysis based on KEGG database (http://www.genome.jp/kegg/). These metabolites could be classified according to the pathways they involved or the functions they performed [25].

### RNA-seq analyses

The obtained samples with mock and ABA treatment were used for RNA extraction. Three biological replicates were performed. The sequencing experiment used a chain specific library to construct the library and performed Illumina platform sequencing. After total RNA extraction, remove rRNA and enrich the target fragment. After second strand cDNA synthesis and adaptor ligation, cDNA fragments were enriched, purified and sequenced [26]. The clean reads were mapped to the reference genome of *Brassica napus* using ZS11 (http://cbi.hzau.edu.cn/cgi-bin/rape/download_ext).

### Correlation analysis of transcriptomic and metabolomic data

The iPath pathway diagram was generated to visualize whether DEMs and DEGs were up-regulated or down-regulated. iPath metabolic pathway was displayed by visualization tool (iPath 3: interactive Pathways Explorer (embl.de)) [27]. To further observe the changes and associations of metabolites and genes, 5 out of the top 10 metabolites significantly different metabolites (VIP > 1, log_2_FC > 1, and *p* < 0.05) and significantly different genes (log_2_FC > 1, *p* < 0.05 and FPKM > 10) were used to describe a correlation network diagram with *r* > 0.90. The correlation analysis and network plot were drawn by Cytoscape. KEGG pathway was displayed by KEGG mapping tools (KEGG Mapper (genome.jp)).

## Results

### Phenotypical observation of rapeseed under ABA treatment during seed germination stage

**Fig. 1:**
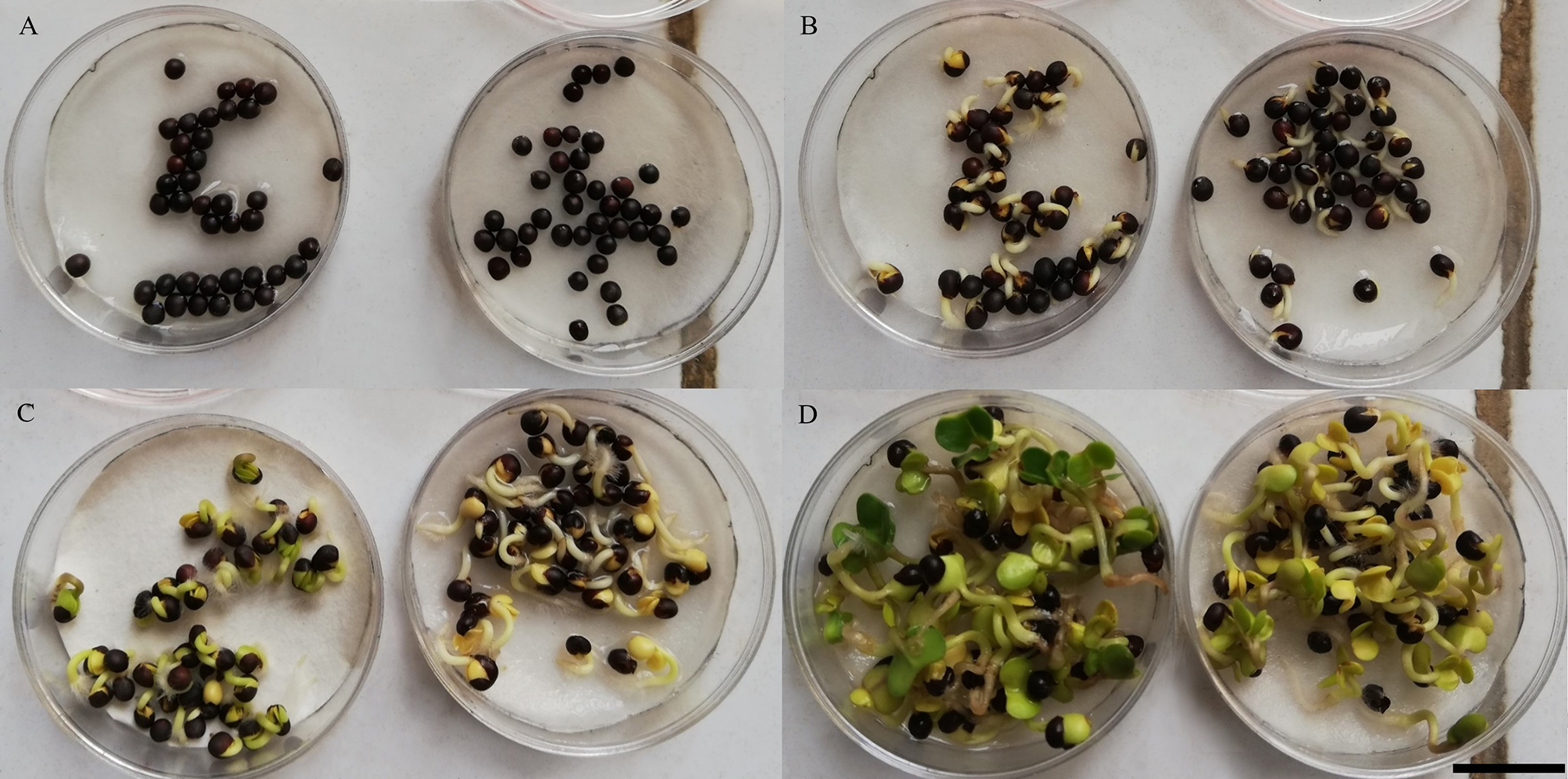
Phenotypic observation of rapeseeds responding to exogenous ABA. (**A**, **B**) 50 seedlings were sown in 35 mm medium, then 1.5 ml of ddH_2_O was added to each medium and put it in a 16/8 h light/dark incubator for 1 day (**A**) and 2 days (**B**) cultivation. (**C**, **D**) Representative images of rapeseed after 1 day (**C**) and 2 days (**D**) mock or ABA treatment. Bar = 1 cm

To investigate the function of ABA on rapeseed during early seedling development, we applied water on petri dish with the seeds of ZS11. After two days cultivation, we found nearly all of the seeds were germinated (Fig. 1A-B). The germinated seeds were sprayed with ddH_2_O or ABA. Both the mock- and ABA-treated seedling were formed cotyledons after 3 days after sowing. However, the cotyledons from 1 day ABA-treated seedlings were gray as compared with mock-treated seedlings (Fig. 1C). As extend cultivation time, all the cotyledons were expanded in both two groups. The cotyledons of seedlings from mock-treated group were turn green while the cotyledons of seedlings from ABA-treated group were still gray (Fig. 1D). These results suggest that ABA mainly inhibits chlorophyll synthesis during early seedling development.

### Metabolome profiles in rapeseed under ABA treatment

**Fig. 2:**
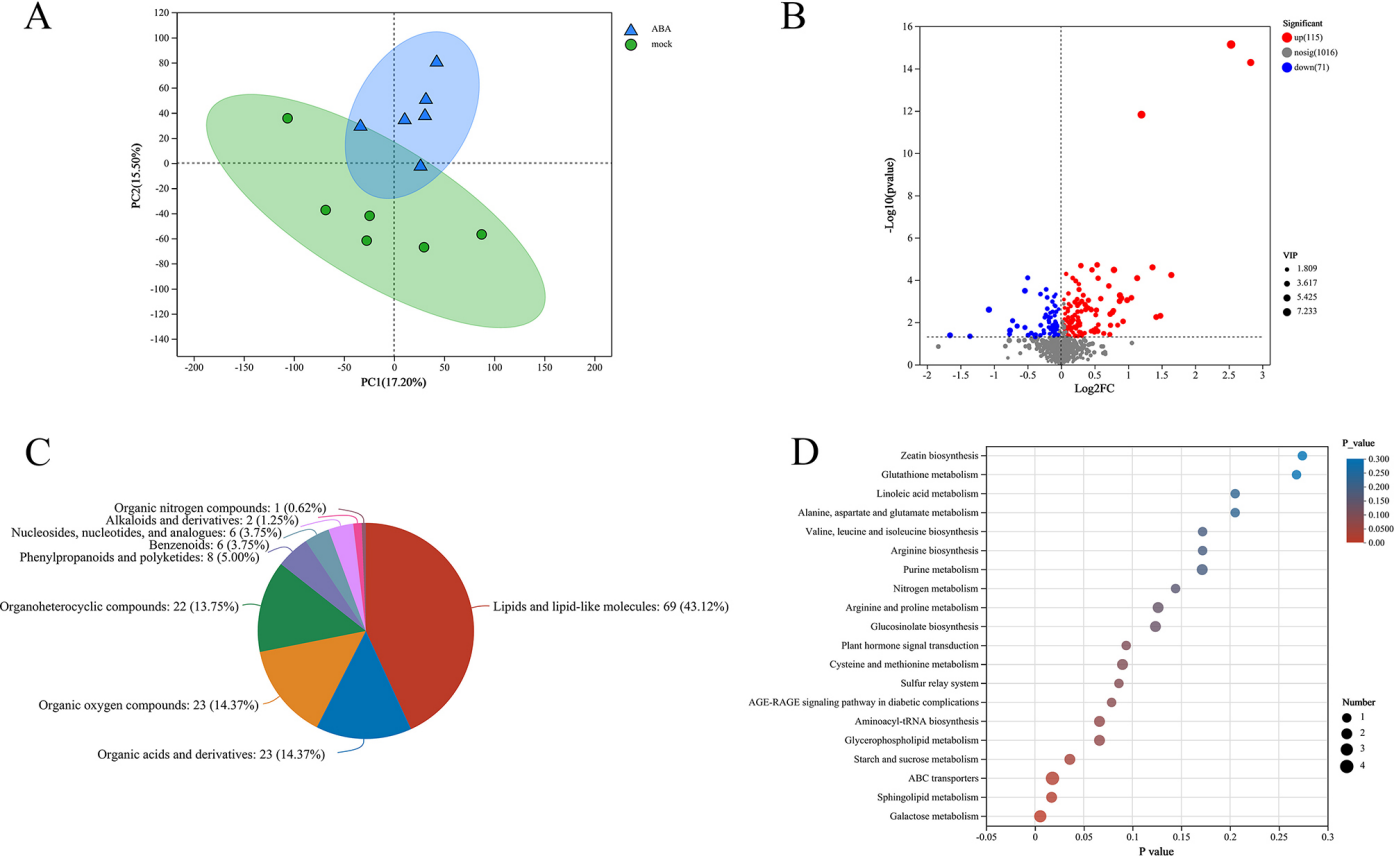
Metabolic profiling of rapeseed in response to exogenous ABA. (**A**) Principal component analysis (PCA) of metabolites identified by mock and ABA-treated samples. (**B**) Volcano plots for differentially expressed metabolites (DEMs) between mock and ABA-treated samples. (**C**) The categories of DEMs. (**D**) Kyoto Encyclopedia of Genes and Genomes (KEGG) pathway enrichment analysis of DEMs

To gain a complete view of rapeseed in response to exogenous ABA during germination, widely targeted LC-MS based metabolomics analysis was performed. A principal component analysis (PCA) of the metabolomic profiles was conducted to provide information about distinctly separate groups (Fig. 2A). The first and second principal components explain 17.20% and 15.50% of the variance among samples, respectively (Fig. 2A). Metabolic profiles under ABA treatment were clearly distinguished by the Partial Least Squares Discriminant Analysis (PLS-DA) score plot (Fig. S1). To verify whether ABA treatment is efficiency, we compared the (+)-ABA (natural synthesis) and (±)ABA (artificial synthesis) levels of metabolites between mock- and ABA treatment. Compared with mock treatment, the (+)-ABA and (±)ABA were 2.30 and 5.80 fold in ABA treatment (Fig. S2). It indicated that the exogenous ABA was absorbed by rapeseed. Then differentially expressed metabolites (DEMs) were screened by a screening standard of *P* < 0.05, VIP > 1, and FC > 1. There were 186 metabolites were detected and showed in the volcano plots (Fig. 2B). Compared with mock-treated samples, there are 115 up-regulated and 71 down-regulated in ABA-treated samples, respectively (Table S1). To further investigate the expression profile of metabolites under ABA-treated, we mainly focus on these core DEMs with log_2_FC > 1 (Table S2). The DEMs mainly belonged to “lipids and lipid-like molecules” (Fig. 2C; Fig. S3). Beside ABA, 9-Hydroxy-4-methoxypsoralen 9-glucoside showed a very significant difference with high VIP, which indicated that carbohydrates metabolism was important for ABA response (Fig. S4). Then a heatmap with hierarchical clustering analysis of proportional content for all DEMs was generated. All DEMs were separated into 9 subclasses, about 2/3 of the DEMs were highly expressed in the ABA-treated groups (Fig. S5).

To identify the significant changes of metabolites under ABA-treated, multivariate analysis was performed. KEGG pathway analysis of DEMs was performed to identify significantly enriched metabolic pathways (Fig. 2D; Table S3). It was found that except that ABA transport pathway, the main enriched pathways were galactose metabolism, starch and sucrose metabolism, sphingolipid metabolism, glycerophospholipid metabolism, cysteine and methionine metabolism, arginine and proline metabolism. These outcomes indicate that exogenous ABA mainly regulates the metabolism of some carbohydrates and lipids.

### Identification of ABA-responsive genes by transcriptome

**Fig. 3:**
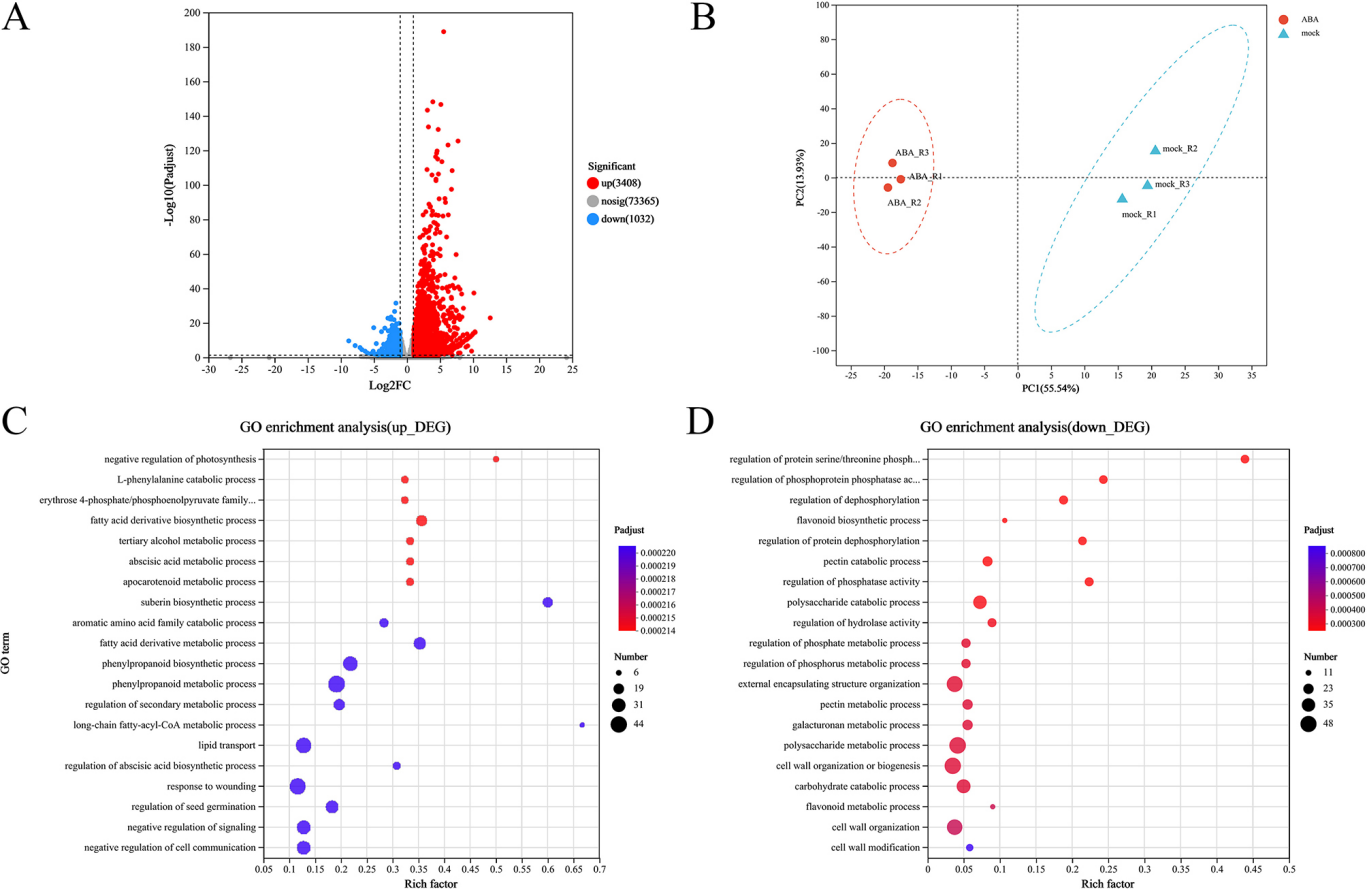
Transcriptomic profiling of rapeseed in response to exogenous ABA. (**A**) Volcano plots showing the number of DEGs between mock and ABA-treated seedlings. (**B**) Principal component analysis (PCA) of mock and ABA-treated samples. (**C**, **D**) Gene ontology-biological process (GO-BP) enrichment analysis based on up-regulated DEGs (**C**) and down-regulated DEGs (**D**)

To screen out the ABA-responsive genes, RNA-seq analysis of rapeseed were performed. The 4,440 differentially expressed genes (DEGs) were identified between mock and ABA treated seedlings (Fig. 3A) (log_2_FC > 1, p_adjust < 0.05). PCA of the transcriptomic data was clearly separated the ABA treatments from the mock treatment based on PC1, with PC1 contributing 55.54% variation and PC2 contributing 13.93% variation (Fig. 3B). These results suggested that a considerable proportion of the transcriptomic changes in rapeseed responded to ABA treatment.

To gain insight into biological processes in response to exogenous ABA, we analyzed gene ontology-biological process (GO-BP) terms enrichment of these 4,440 DEGs (Table S4). The highly enriched DEGs are associated with the phenylpropanoid biosynthetic process, regulation of seed germination, secondary metabolic process and seedling development. The most enriched GO terms were related to phenylpropanoid metabolism, which in response to phytohormones and biotic/abiotic stresses (Table S5). Interestingly, hormone-related GO terms include ABA metabolic process and apocarotenoid metabolic process, while the phenylpropanoid metabolism are not enriched in the top 20 metabolic pathways. These results suggested that ABA plays an important role in seed germination. We further conducted GO-BP enrichment analyses of up-regulated and down-regulated DEGs, respectively (Fig. 3C-D). Among up-regulated transcripts under ABA treatment, significant enrichments were observed in GO-BP terms, such as “negative regulation of cell communication”, “lipid transport”, “response to wounding”, “fatty acid derivative metabolic process”, “negative regulation of signaling”. Importantly, the up-regulated DEGs were largely enriched in “phenylpropanoid metabolic” and “phenylpropanoid biosynthetic” processes. Our GO terms results were highly consistent with previous publication, many candidate genes were related to “response to stimuli”, “response to oxygen-containing compounds”, “lipid response”, as well as “transport and seed dormancy processes” in brassica during seed germination [28]. As significant enrichment in down-regulated DEGs was observed in GO-BP terms related to “cell wall organization or biogenesis”, “external encapsulating structure organization”, “polysaccharide metabolic process”. The results showed that ABA plays an important role in cell formation. In addition, the “carbohydrate catabolic” process was also enriched. The data also supports our finding that carbohydrates were important metabolites in the ABA response at metabolome levels.

### Correlation and integrative analysis between metabolome and transcriptome

**Fig. 4:**
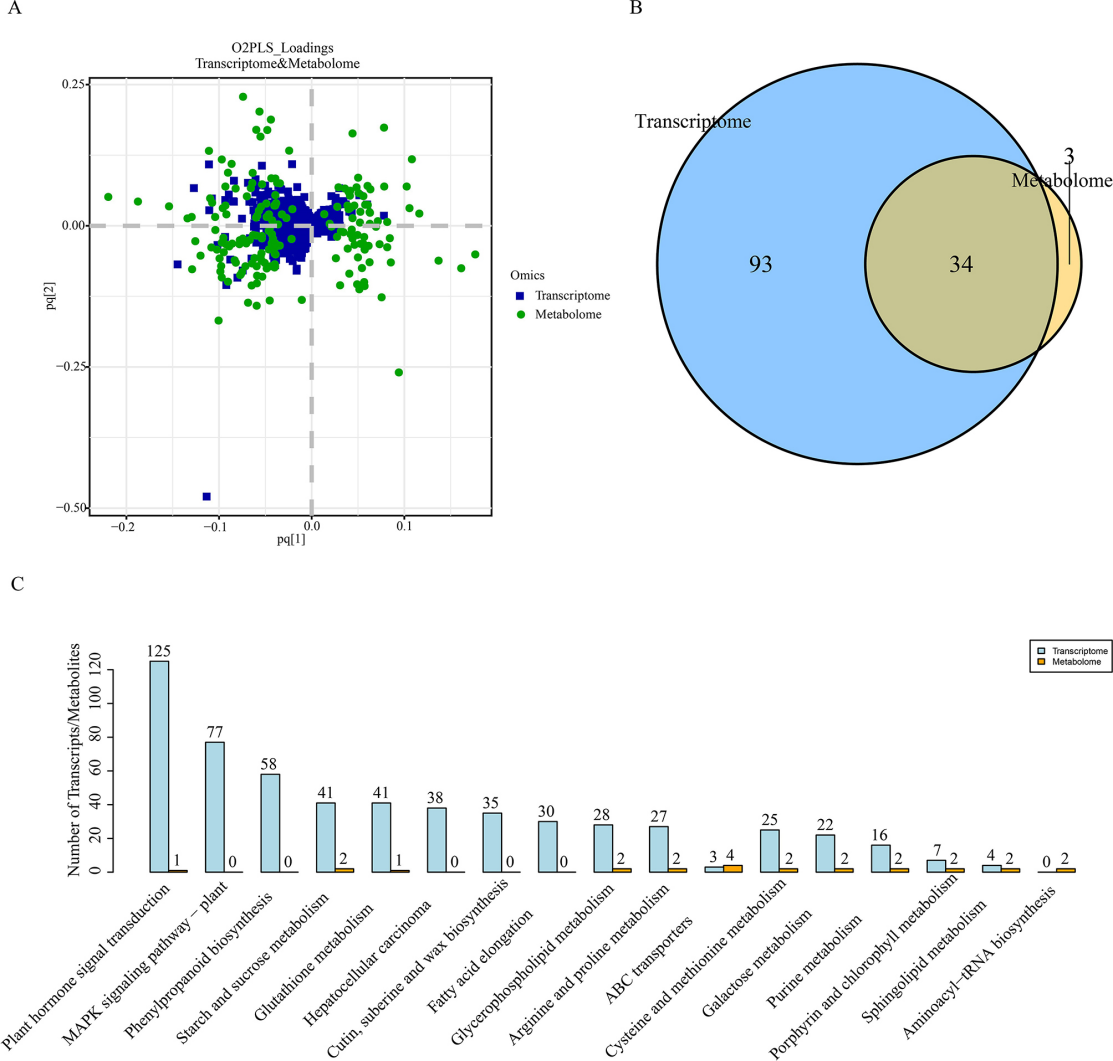
Correlation analysis between metabolomic and transcriptomic data. (**A**) O2PLS loading plots identifies joint variation between transcript and metabolite dataset. The horizontal and vertical coordinates represent the joint load value, p-value represents the gene load value and q-value represents the metabolite load value. (**B**) Venn diagram of DEGs and DEMs involved in the KEGG pathway. (**C**) The top 10 KEGG pathways with the highest number of DEGs and DEMs identified by trancriptome and metabolome

To further understand the correlation between ABA-responsive metabolome and transcriptome, the loading values from DEMs and DEGs were used for showing the overlap of these two omics. This analysis suggested that these two omics showed highly correlation (Fig. 4A). The Venn diagram also showed that almost all the pathways from DEMs were overlapped with the pathways from DEGs (Fig. 4B). DEMs almost overlapped all pathways which were involved in DEGs (Fig. 4C, Table S5). DEMs and DEGs were mainly enriched in “starch and sucrose metabolism”, “glycerophospholipid metabolism”, “arginine and proline metabolism”.

**Fig. 5:**
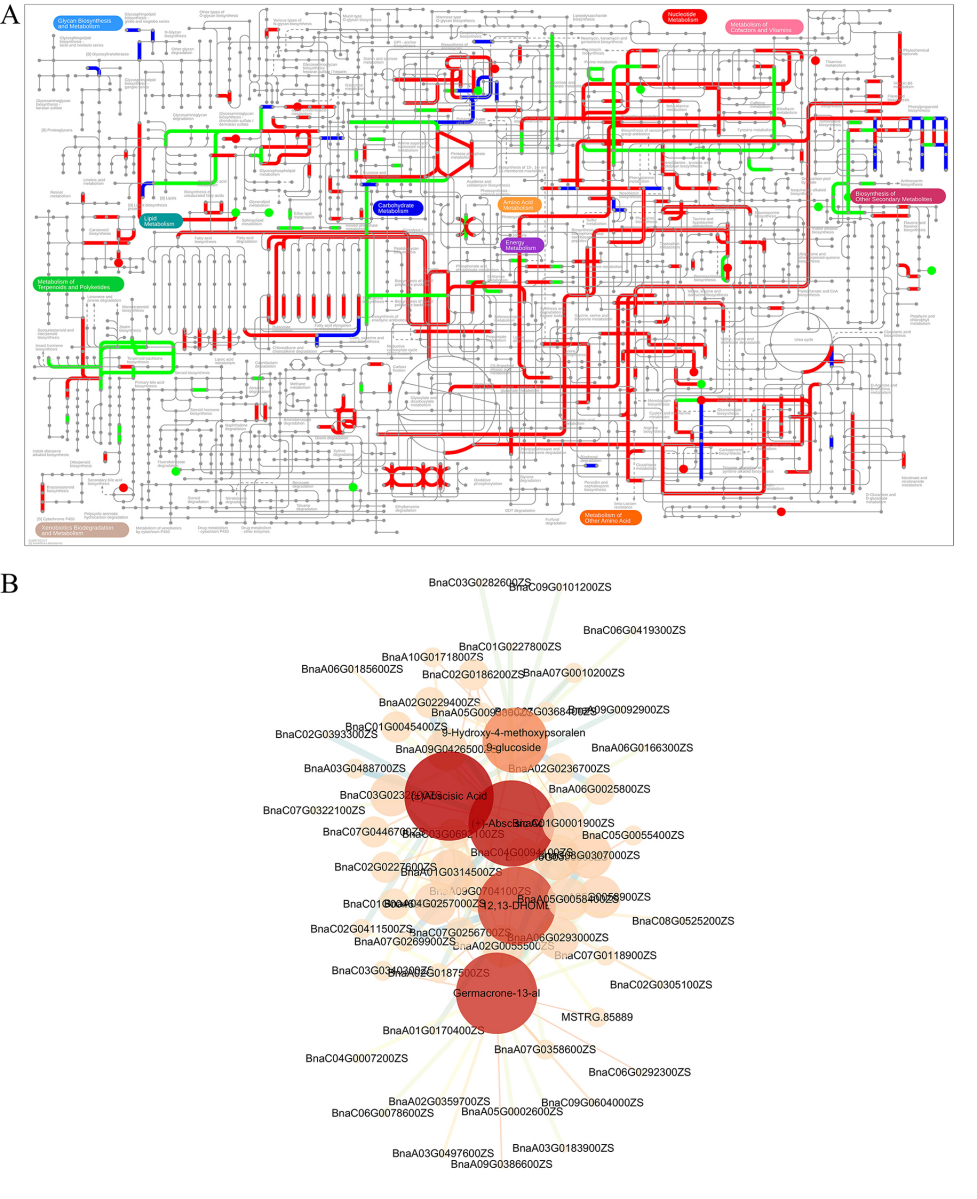
Integration analysis of DEGs and DEMs. (**A**) The iPath analysis of DEGs and DEMs revealed the metabolic network of differentially expressed metabolites and corresponding metabolic pathways throughout the entire KEGG biological system. Red dots and green dots represent differentially expressed metabolites that are upregulated and downregulated, respectively. The red line and green line represent the pathway annotated by up-regulated and down-regulated genes, respectively, and the blue line represents the pathway annotated by genes in both gene sets. (**B**) Connection network between five metabolites in top10 DEMs and screened DEGs (log_2_FC > 1, *p* < 0.05, and FPKM > 10). The size of nodes represents the number of degrees. Edges represent relationships (p-value) between DEMs and DEGs

Considering metabolite abundances were determined by transcript levels, we also conducted a correlation analysis between the transcriptome and metabolome. Through iPath analysis, the DEGs and DEMs were associated with “carbohydrate metabolism”, “lipid metabolism”, “biosynthesis of other Secondary metabolism” and “energy metabolism” (Fig. 5A; Table S6). To investigate the robust changes of metabolites, we carried out a correlation test between the core altered DEMs and major DEGs. The interaction network for was performed between 5 DEMs in top 10 and 131 DEGs (Fig. 5B; Table S7). The network showed that these DEMs were clustered together and shared common DEGs. Especially, ABA correlated with more DEGs than other metabolite. In summary, the correlation analysis suggested that these DEGs might play a direct or in direct regulatory role in key DEMs metabolism.

### Starch and sucrose metabolism under ABA treatment

**Fig. 6:**
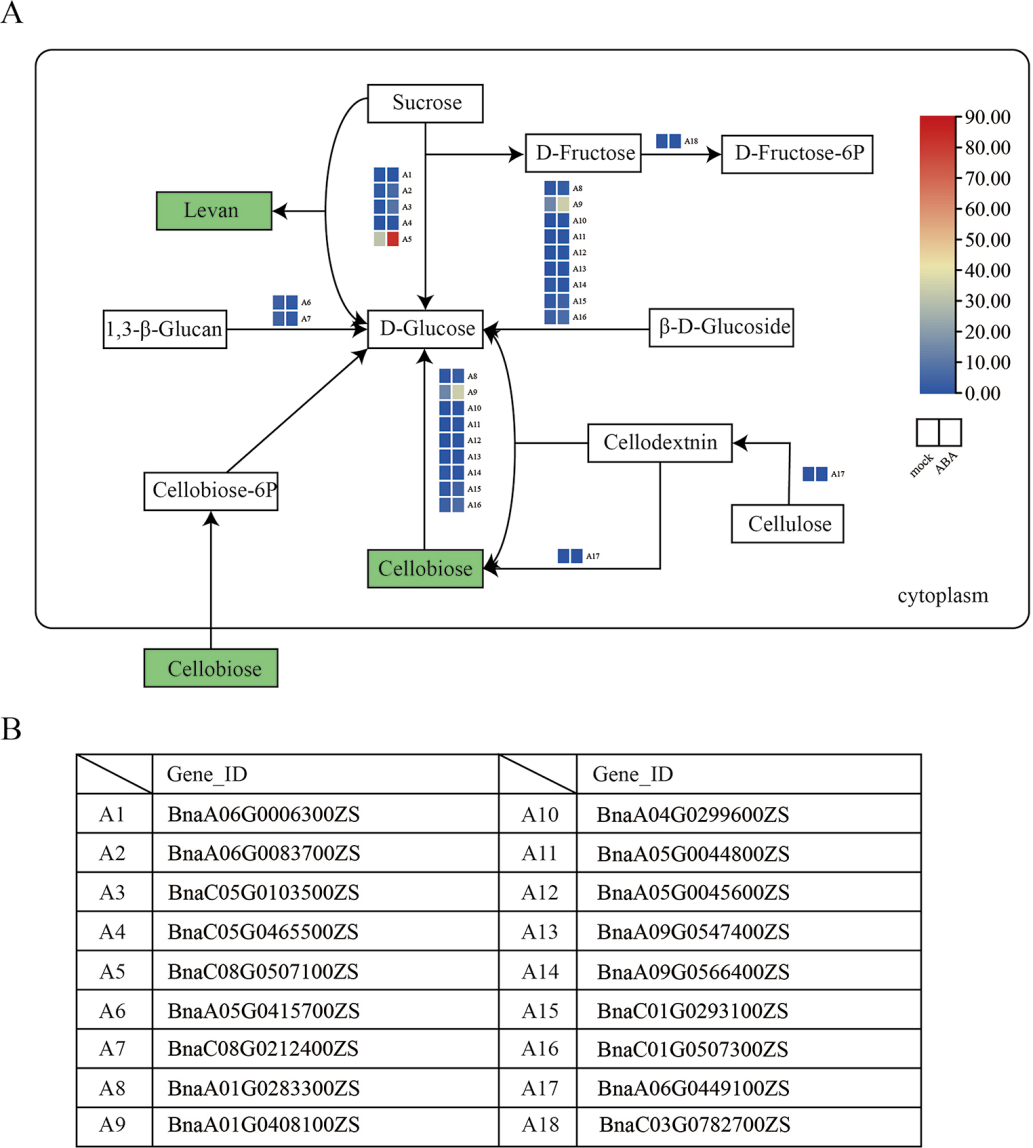
Expression profiles of DEGs and DEMs associated with starch and sucrose metabolism. (**A**) The regulatory network of starch and sucrose metabolism were revealed by DEGs and DEMs. (**B**)The genes were involved in levan and cellobiose metabolism

To further explore transcriptional regulations under ABA-treated in rapeseed, major DEGs that were highly correlated with the top 10 DEMs and DEGs in the comparison (mock vs. ABA) were assigned (Fig. 4C). According to the top 10 DEMs and DEGs, the highly related DEGs assigned to the starch and sucrose metabolism pathways (map00500) were further investigated. This result also supported our finding that DEGs and DEMs were enriched in carbohydrate metabolism in iPath analysis. It showed the proposed general layout of starch and sucrose metabolism and relative changes of DEMs under ABA-treated (Table S8). The two metabolites, levan and cellobiose were found significantly downregulated. Enzymes closely involved in cellobiose formation and metabolism include sucrose, trehalose and D-Glucose. These evidences indicated that the exogenous ABA might regulates starch metabolism by inhibiting the synthesis of cellobiose. The expression patterns of the DEGs which were involved in the starch and sucrose metabolism pathway were summarized (Fig. 6). These DEGs were highly correlated with mentioned DEMs.

## Discussion

ABA is one of the crucial phytohormone affecting the seed development and stress resistance of plant species [8]. Although many genes involved in the ABA signaling pathway have been documented in numerous studies [10, 29], the regulatory mechanisms concerning suppression by ABA of seed germination and post-germination growth are largely understood [13]. Studying the changes in seeds after applying exogenous ABA during seed germination can not only help us understand the mechanism of ABA action in seed germination, but also be used for subsequent research on post-germination growth. Therefore, it is important to use high-throughput omics approaches to identify ABA-responsive genes or metabolites. In this current study, an integrated analysis of transcriptomes and metabolomes under ABA-treated was carried out in ZS11, and a total of 4440 significant DEGs and 186 DEMs were identified. These data allowed us to gain insights into the key metabolites, genes, and metabolic pathways involved in ABA response during rape seed germination. KEGG analysis of DEMs showed that ABA transport was significantly enriched under exogenous ABA. Similar results were also reported in Arabidopsis [30]. Multiple sites of ABA biosynthesis and ABA transporters have been reported in previous studies [9, 31]. These results revealed that ABA transport is a key regulatory for exogenous ABA to play a role in rapeseed seed germination. Besides ABA transport, the metabolism of some sugars, lipids, and amino acids were also regulated by exogenous ABA. These results were consistent with the previous studies in quinoa and Fagaceae [32]. Especially, sugar is the most important carbohydrate reserve in plants, which can be divided into soluble sugar and insoluble sugar according to their solubility in water. Soluble sugar mainly includes glucose, fructose, sucrose, maltose, stachyose and raffinose, while insoluble sugar mainly includes starch. In the process of seed germination, starch decomposes to provide energy for seed germination and initial growth, the content of starch mainly shows a decreasing trend in other plants [33]. The content of soluble sugar has two types of expression patterns. In starch seeds, such as sorghum, the content of soluble sugar increases during germination [34]. As oil seed, the soluble sugar content of rapeseed seeds decreases at first and then increases. In our study, starch and sucrose metabolism were down-regulated. Therefore, exogenous ABA may inhibit seed germination by inhibiting starch decomposition and energy release. Lipids are not only the foundation of cell membrane boundaries and compartmentalization functions, but also participate in signal transduction and hormone production. Sphingolipids and their metabolites are a major component of these membranes [35]. The lipid metabolism pathway has been developed in previous studies [36]. Sphingolipid and glycerophospholipid all enriched based on DEMs. It suggested that membrane synthesis may be another pathway of exogenous ABA action. These results revealed that the inhibition of exogenous ABA on seed germination is mainly reflected in the metabolism of sugar, lipids, and amino acids. The glucosinolate biosynthesis, nitrogen metabolism and sulfur relay system were enriched. Glucosinolates (GSLs), found mainly in species of the Brassicaceae family, are one of the most well-studied classes of secondary metabolites and GSLs were sulfur- and nitrogen- containing secondary metabolites functioning in plant defense [37]. Interestingly, S-containing compounds like cysteine (Cys), methionine (Met) and Glutathione (GSH) were all enriched, suggested that Sulfur metabolism in seeds may has immense importance in the role of exogenous ABA during the time of seed germination [38]. Obvious differences were shown among DEMs (Table S1). These major metabolites changed may be mainly responsible for the germination inhibition.

Transcriptome data indicated that of the clusters of down-regulated genes, some related to the formation of cell, such as the clusters of cell wall organization or biogenesis, external encapsulating structure organization and polysaccharide metabolic process. Among them, polysaccharides are almost present in the cell walls of all terrestrial plants [39]. The cell wall is composed of cellulose, hemicellulose, pectin, and lignin. Cellulose, consisting of unbranched β-(1,4)-linked glucan chains, is the main polymer in most plant cell walls [29]. As previously studied, the biomechanics of embryo cell growth during seed germination depend on irreversible cell wall loosening [17]. We can speculate that exogenous ABA ultimately inhibits seed germination by regulating genes related to cell wall formation. Similarly, previous studies have shown that ABA can inhibit the rupture of endosperm of brassicaceae species, and endosperm is the barrier of radicle protrusion of many angiosperm seeds. Gibberellin stimulates germination by regulating the abundance of cell-wall-remodeling enzymes (CWREs) [40]. GO-BP enrichment analysis of up-regulated genes showed that phenylpropanoid pathways was enriched. Previous studies also reported that ABA play a directed or indirect role in the induction of phenylpropanoid biosynthesis [41]. Similar results were also reported in cotton [42]. They found 9 proteins involved in phenylpropanoid biosynthesis were upregulated after ABA treatment, suggesting that this pathway might play important roles in the response to ABA. Flavonoids, as phenylpropanoid compounds, also have functions such as regulating growth and stresses responses [43]. Different from phenylpropanoid, flavonoid biosynthetic process and metabolic process were enriched in down-regulated genes.

Integrative analysis of the transcriptomes and metabolomes showed that DEGs and DEMs were associated with mostly the “carbohydrate metabolism”, “lipid metabolism”, “biosynthesis of other secondary metabolism” and “energy metabolism”. Combined with the association analysis of DEMs and DEGs, we screened out “starch and sucrose metabolism” pathways. Carbohydrates are an important source of cellular energy. Therefore, a good carbohydrate supply is closely related to seed vitality and germination process [33]. Research has shown that seedlings adapt to stress by retaining starch and retarding growth through both ABA-dependent and -independent pathways [44]. The previous KEGG pathway analysis showed that levan and cellobiose, two metabolites in the starch and sucrose pathways, were significantly down regulated. Cellobiose, a β-1,4-linked glucose dimer, is a major cellodextrin of the enzymatic hydrolysis (via endoglucanase and exoglucanase) of cellulose. Studying the degradation of cellulase has the potential to turn cellulose into a new fuel and produce various industrial products [45]. Cellulose is the main component of most plant cell walls [29], which is completely hydrolyzed to glucose by a cellulase cocktail of three different enzymes endoglucanase, exoglucanase (including cellodextrinase and cellobiohydrolase) and β-glucosidase. The rate limiting step of cellulase hydrolysis to glucose is β-glucoside hydrolyzes cellobiose into two glucose monomers [46]. Recently, it has been found that cellobiose phosphorylase can provide energy advantage for Cellobiose degradation through phosphorylation pathway [47]. The inhibition of cellulase and β-glucosidase activity led to a significant decrease in cellobiose content. The results showed that exogenous ABA regulated the hydrolysis of cellulose into glucose, especially the hydrolysis of cellulose into cellobiose, thereby affecting the loosening of cell wall and ultimately inhibiting seed germination.

Levan is a sugar polymer composed of fructose with β-(2–6)-linkages, having β-(2−1) -connected fructose side chains. Levan can be produced by plants and micro-organisms, Plant fructans generally have a low degree of polymerization (DP < 10^1^ to 10^2^) [48]. Levan are synthesized by the action of enzyme levansucrase (EC 2.4.1.10) utilizing sucrose as the only carbon source [49]. The significant downregulation of levan content in the pathway may be due to the inhibition of ABA on starch degradation into levan pathway. Fructan can be divided into three different types: (1) Inulin type, which is composed of β-(2−1) Fructosyl bond composition; (2) Levan type Fructan, mainly composed of β-(2–6) Fructosyl unit connection; (3) Mixed Fructan, linked by β-(2–6) and β-(2−1) Composition of fructose based units [50]. Fructan in plants is produced in the early stages of vegetative and reproductive development. In addition to being one of the main stored carbohydrates, fructan also has the function of maintaining membrane stability and free radical scavenging during Abiotic stress [51]. The research shows that the metabolism of Fructan in *Vernonia herbacea* and *Chrysolaena obovata* changes periodically. During seed germination, Fructan decreases as a reserve content to provide energy for growth, and then Fructan content increases in the vegetative growth stage [52]. Fructan is hydrolyzed by fructan exohydrolase (FEH) [53], which exists not only in fructan-synthesizing species, but also in non-Fructan plants [54]. The function has been well studied in the growth and development of Fructan plants [53]. It is generally believed that cell wall invertase (CWI) is the ancestor of FEH [55]. Based on substrate differences, FEHs are classified into three types: (1) 1-FEH mainly hydrolyzes inulin-type fructan; (2) 6-FEH mostly degrades levan-type fructan; (3) 6&1-FEHs degrade mixed types of fructan. Different plant hormones are known to regulate fructan metabolism [56]. There is evidence that ABA regulates fructan metabolism [57]. Similar to previous research in wheat, transcripts for three FTs and for 6-FEH decreased, while transcripts for 1-FEH increased in ABA-fed wheat stems compared to controls [58]. We speculate that exogenous ABA inhibits starch degradation into levan, leading to a significant downregulation of levan content and a decrease in 6-FEH transcripts.

### Electronic supplementary material

Below is the link to the electronic supplementary material.

Supplementary Material 1

Supplementary Material 2

Supplementary Material 3

Supplementary Material 4

Supplementary Material 5

Supplementary Material 6

Supplementary Material 7

Supplementary Material 8

Supplementary Material 9

Supplementary Material 10

Supplementary Material 11

Supplementary Material 12

Supplementary Material 13

Supplementary Material 14

## Data Availability

The raw sequence data reported in this paper have been deposited in the Genome Sequence Archive (Genomics, Proteomics & Bioinformatics 2021) in National Genomics Data Center (Nucleic Acids Res 2022), China National Center for Bioinformation / Beijing Institute of Genomics, Chinese Academy of Sciences (GSA: CRA014177) that are publicly accessible at https://ngdc.cncb.ac.cn/gsa.
