# Supplementary figures and images for: Metabolome and transcriptome analyses reveal changes of rapeseed in response to ABA signal during early seedling development
Source: BMC Plant Biol. 2024 Apr 5;24:245. doi: 10.1186/s12870-024-04918-8 (PMC11000593; doi:10.1186/s12870-024-04918-8)

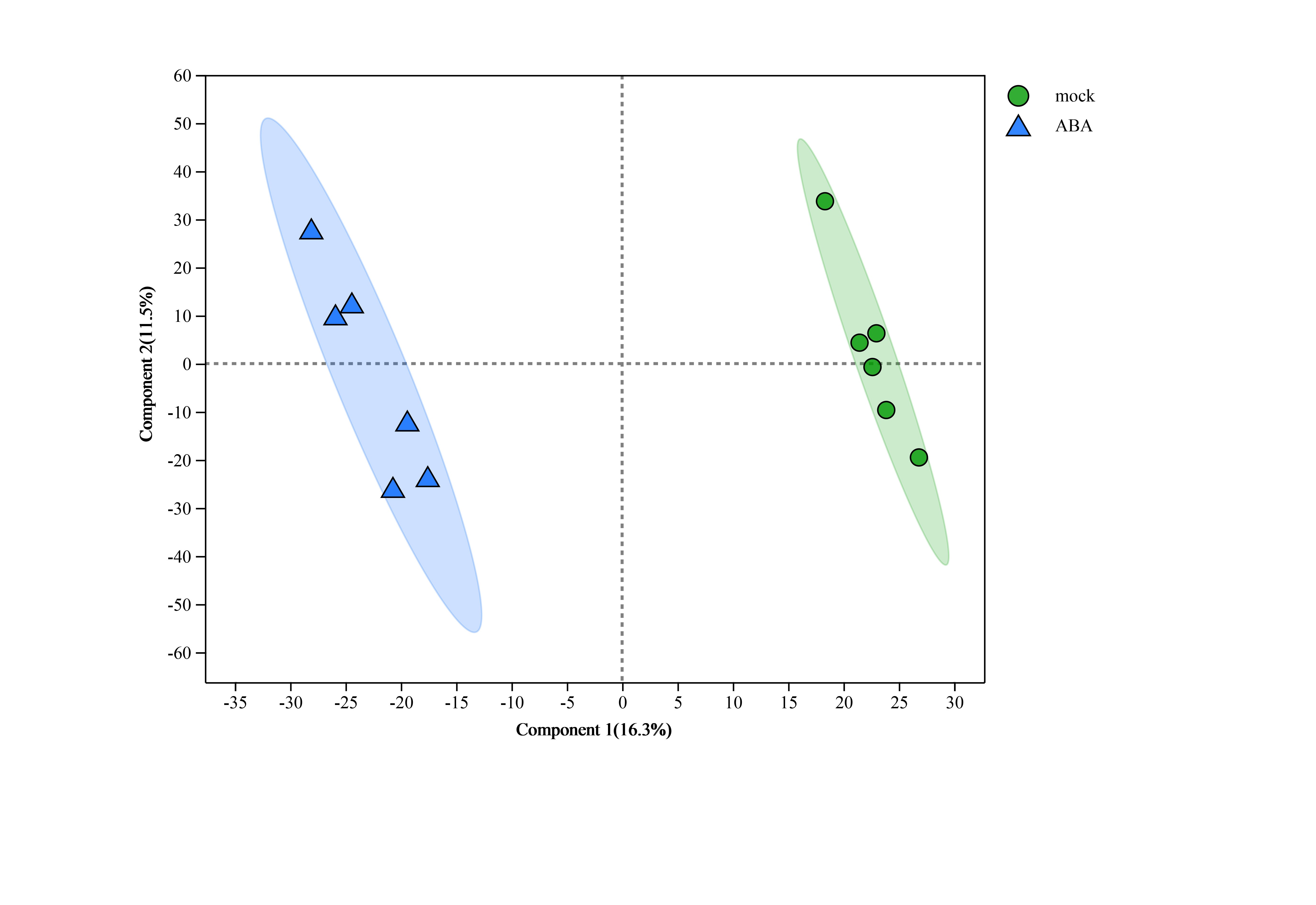

Supplement: Supplementary file 1 — Supplementary Material 1 [file 12870_2024_4918_MOESM1_ESM.jpg]

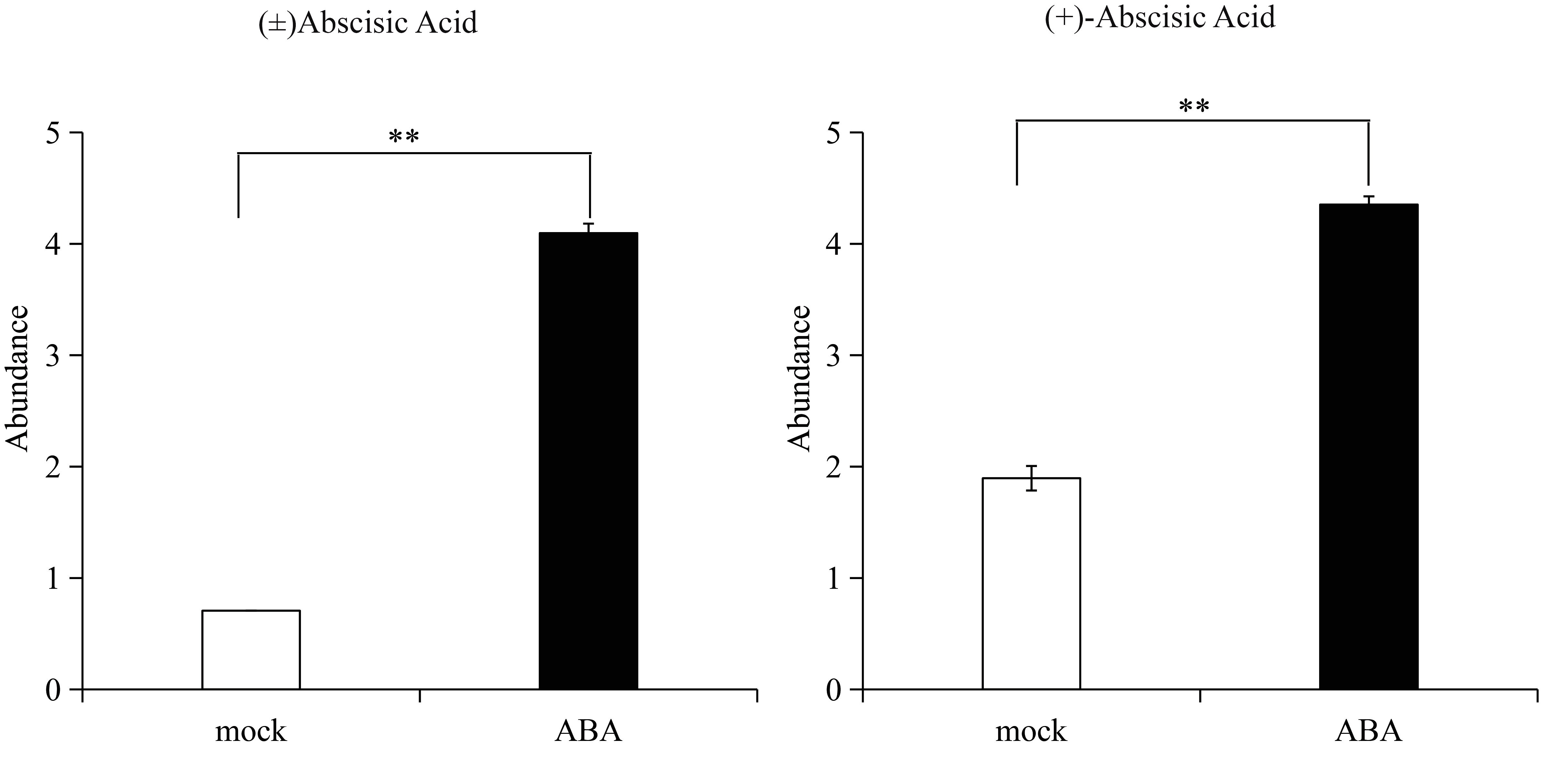

Supplement: Supplementary file 2 — Supplementary Material 2 [file 12870_2024_4918_MOESM2_ESM.jpg]

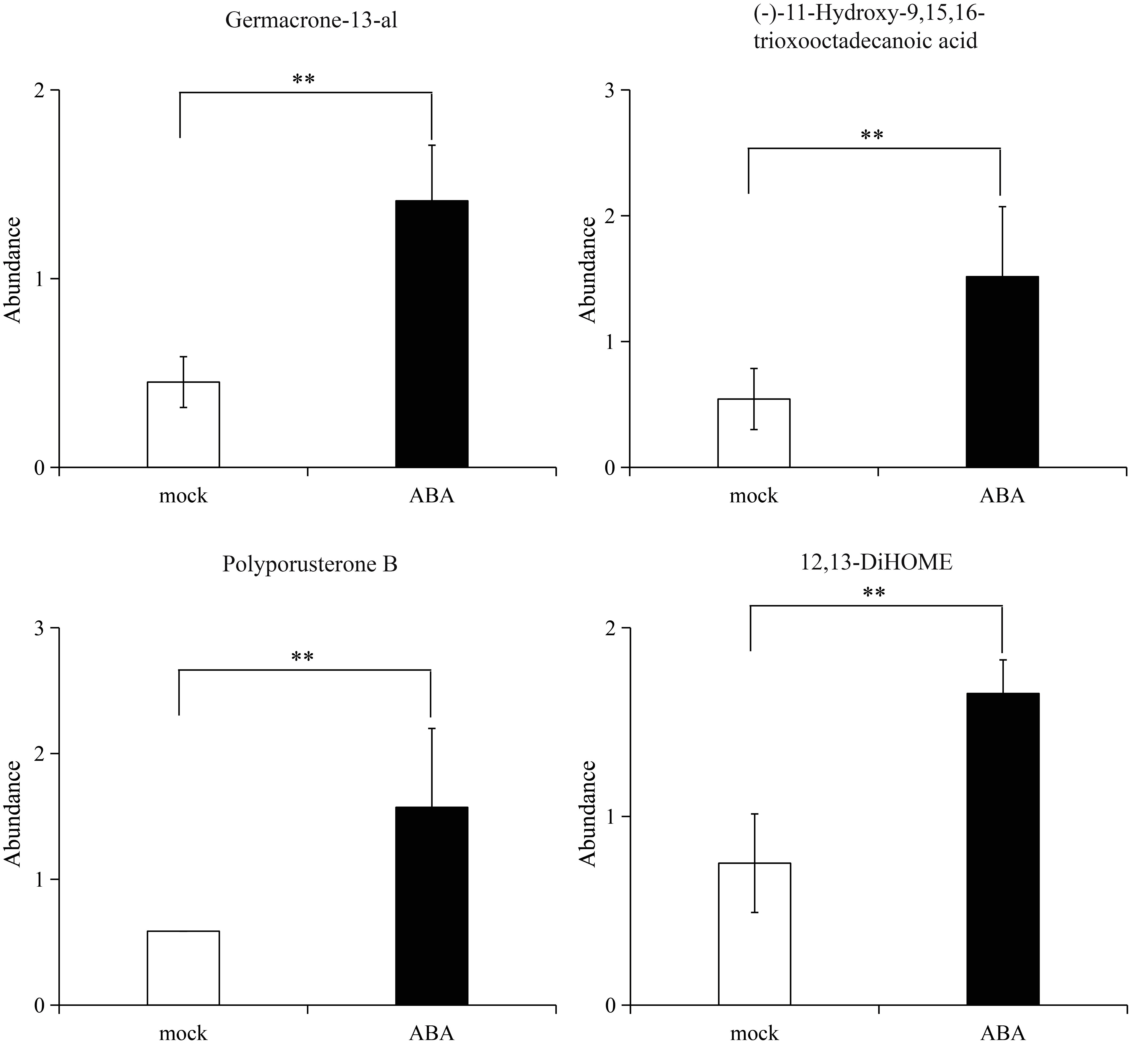

Supplement: Supplementary file 3 — Supplementary Material 3 [file 12870_2024_4918_MOESM3_ESM.jpg]

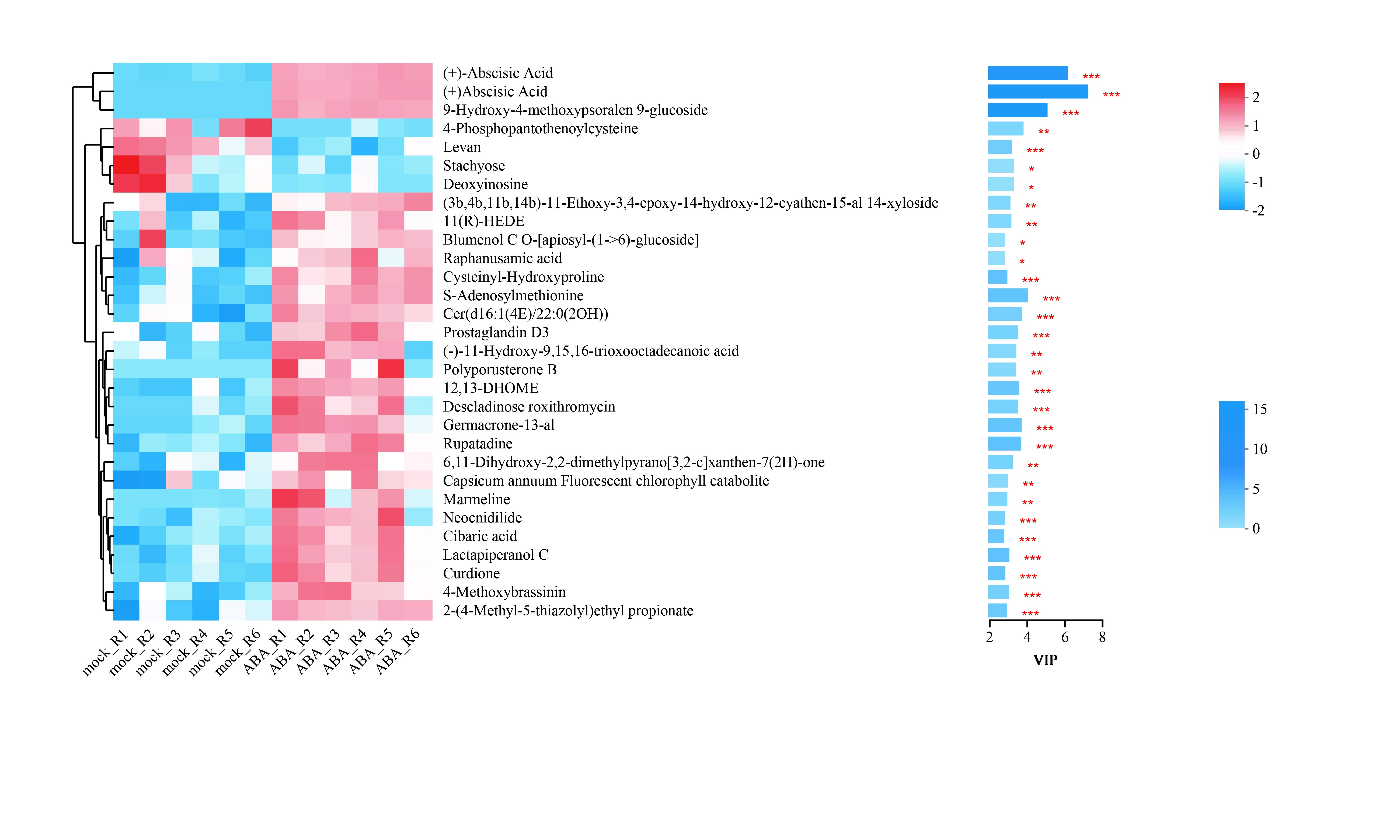

Supplement: Supplementary file 4 — Supplementary Material 4 [file 12870_2024_4918_MOESM4_ESM.jpg]

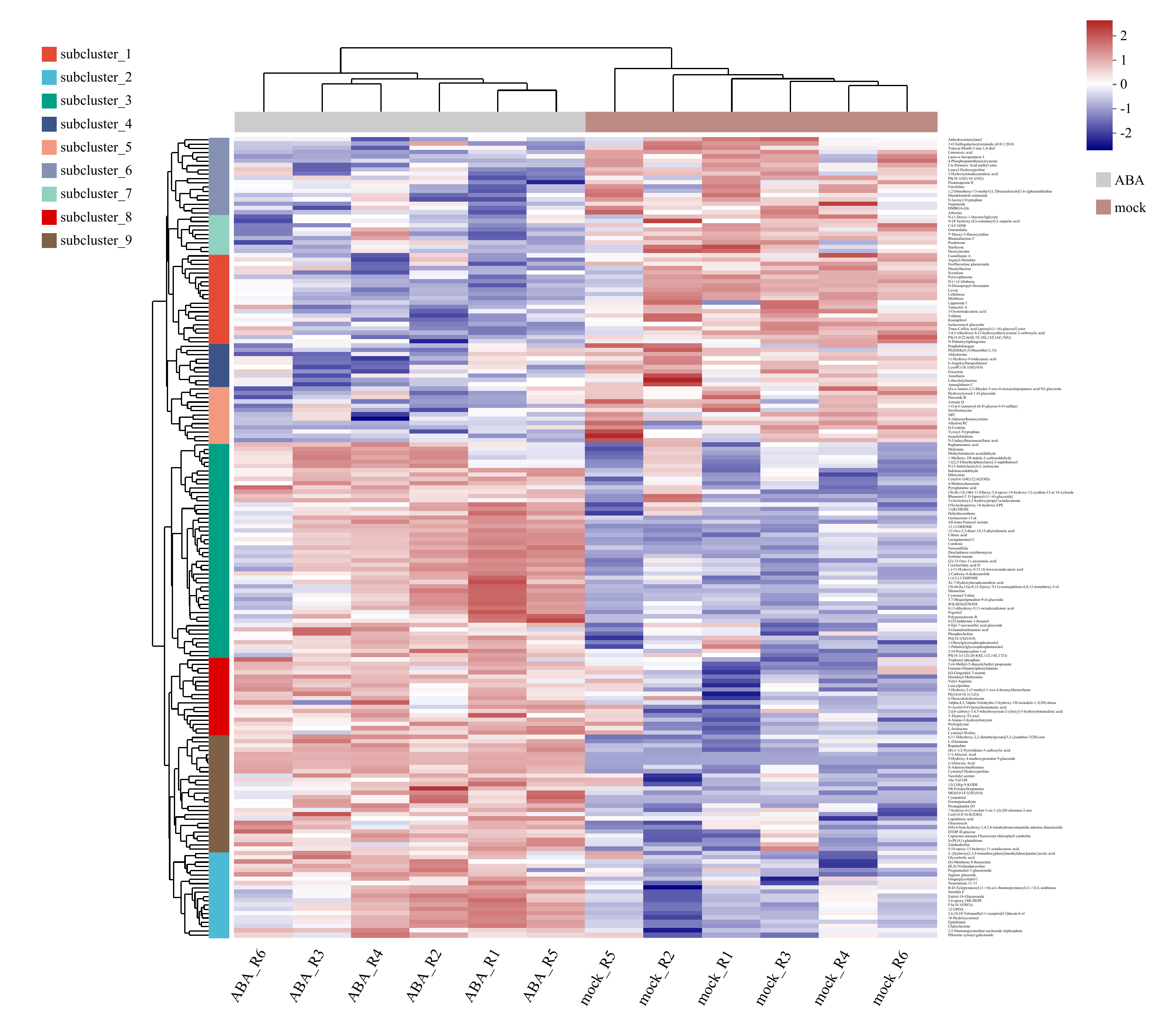

Supplement: Supplementary file 5 — Supplementary Material 5 [file 12870_2024_4918_MOESM5_ESM.jpg]
