# Supplementary material for: Metabolome and transcriptome analyses reveal changes of rapeseed in response to ABA signal during early seedling development
Source: BMC Plant Biol. 2024 Apr 5;24:245. doi: 10.1186/s12870-024-04918-8 (PMC11000593; doi:10.1186/s12870-024-04918-8)
Supplement: Supplementary file 6 — Supplementary Material 6 [file 12870_2024_4918_MOESM6_ESM.docx]

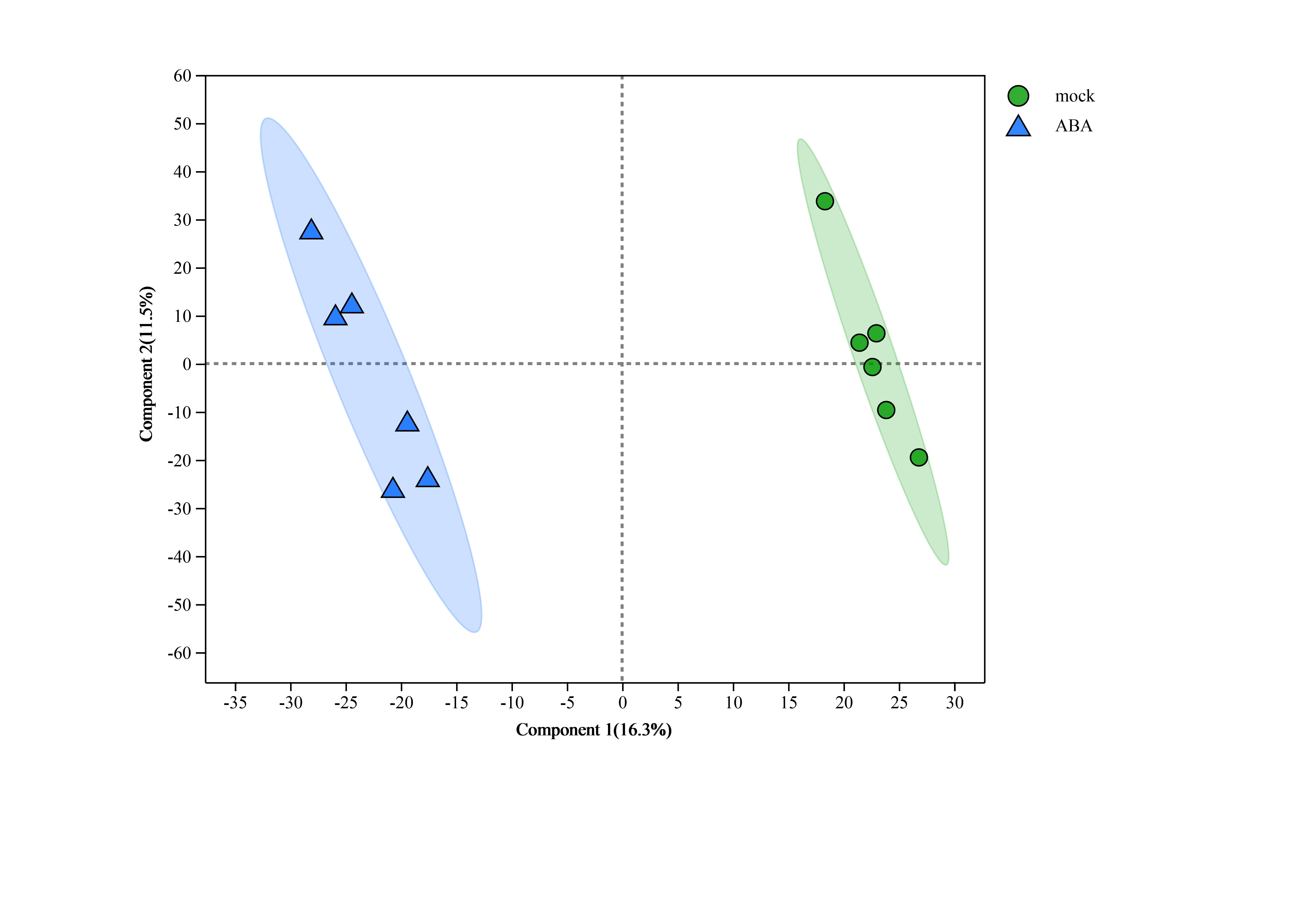


**Fig. S1** Partial Least Squares Discriminant Analysis (PLS-DA) score plot.

PLS-DA of metabolites based on mock and ABA-treated samples.


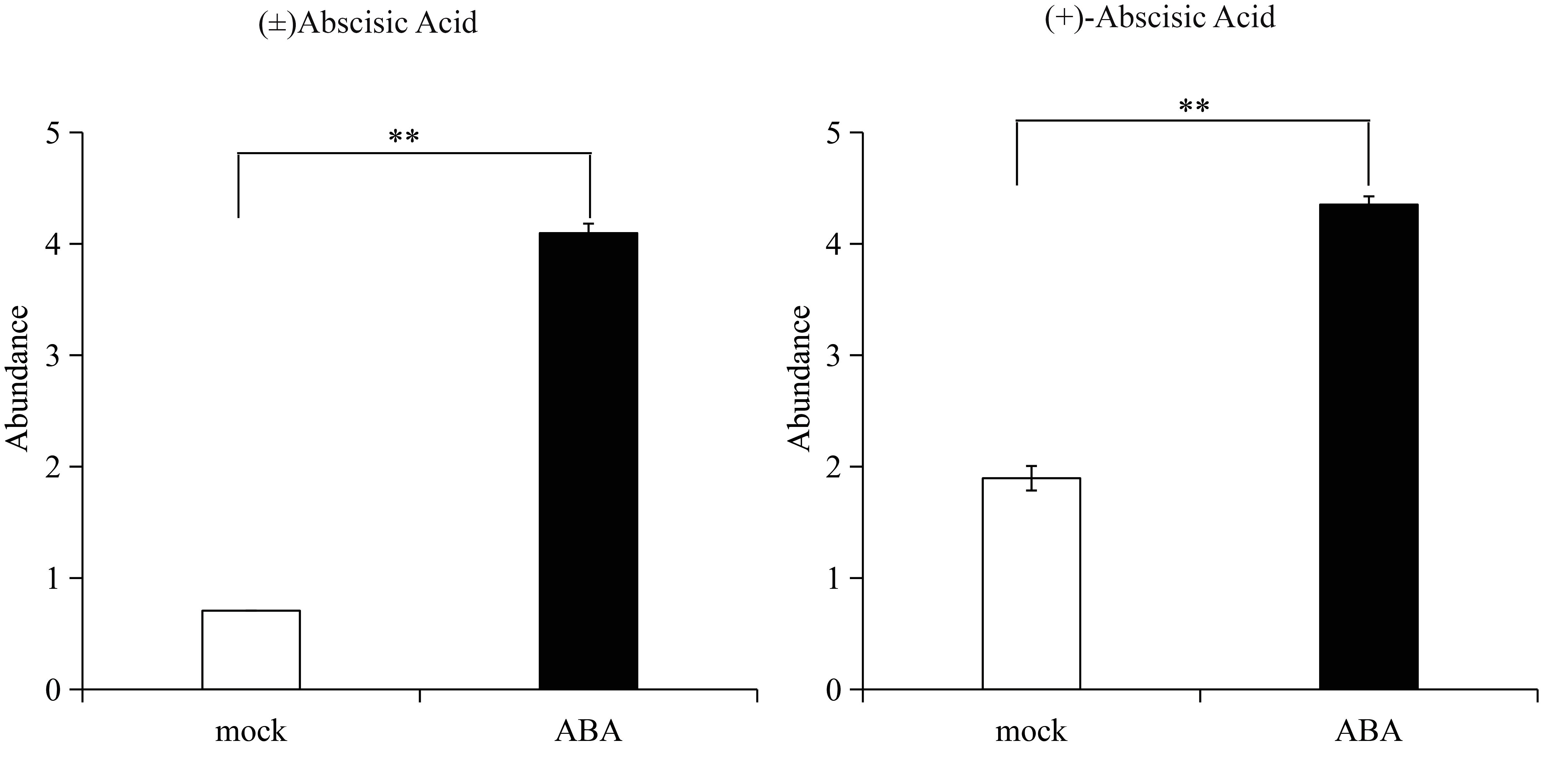


**Fig. S2** The abundance of ABA between mock and ABA-treated rapeseed.

The abundance of (±)ABA and (+)-ABA was compared between mock and ABA-treated samples. ** represents significant differences under p < 0.01.


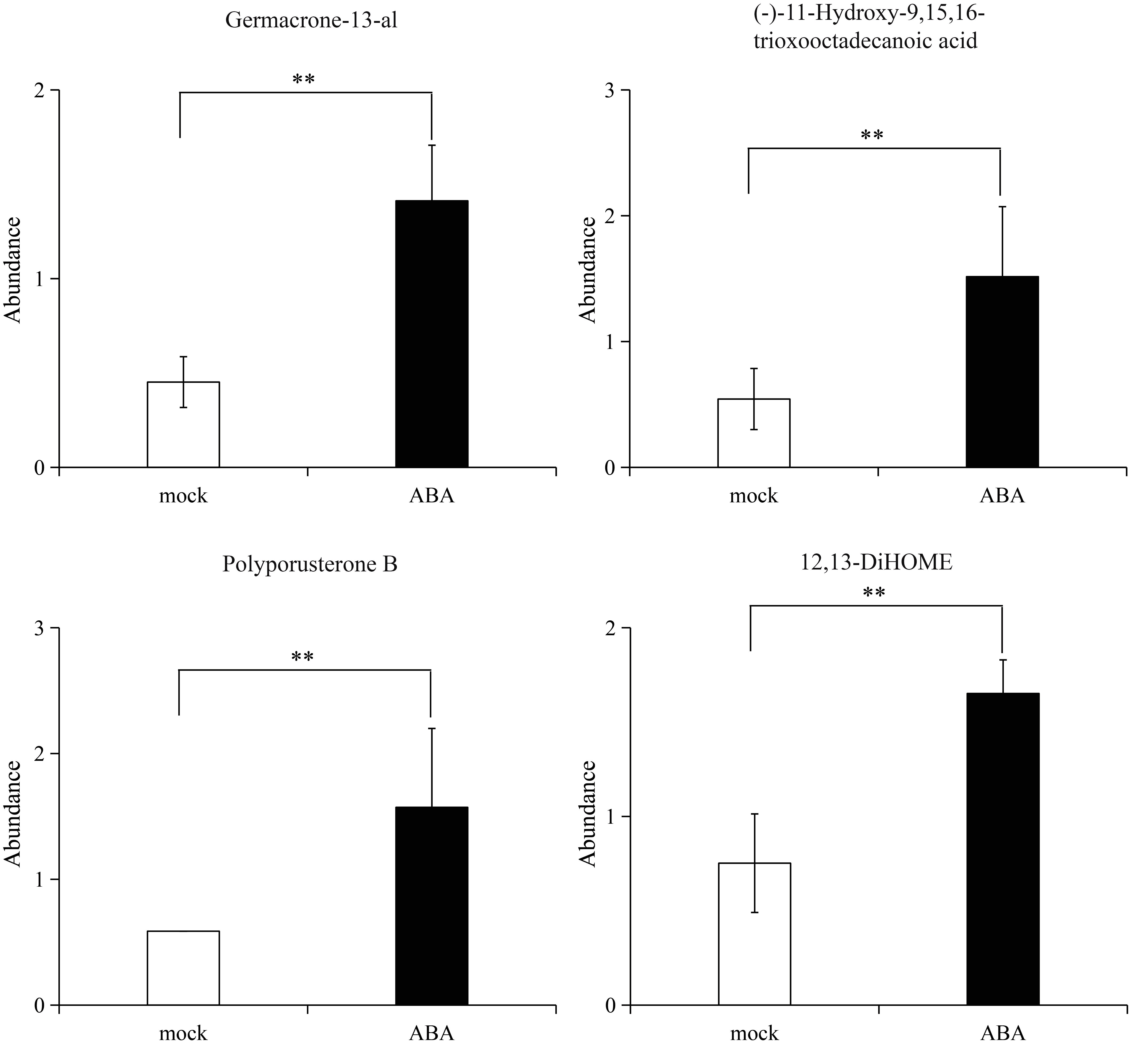


**Fig. S3** The abundance of lipid-related compounds between mock and ABA-treated rapeseed.

The abundance of germacrone-13-al, (-)-11-Hydroxy-9,15,16-trioxooctadecanoic acid, polyporusterone B and 12,13-DHOME was compared between mock and ABA-treated samples. ** represents significant differences under p < 0.01.


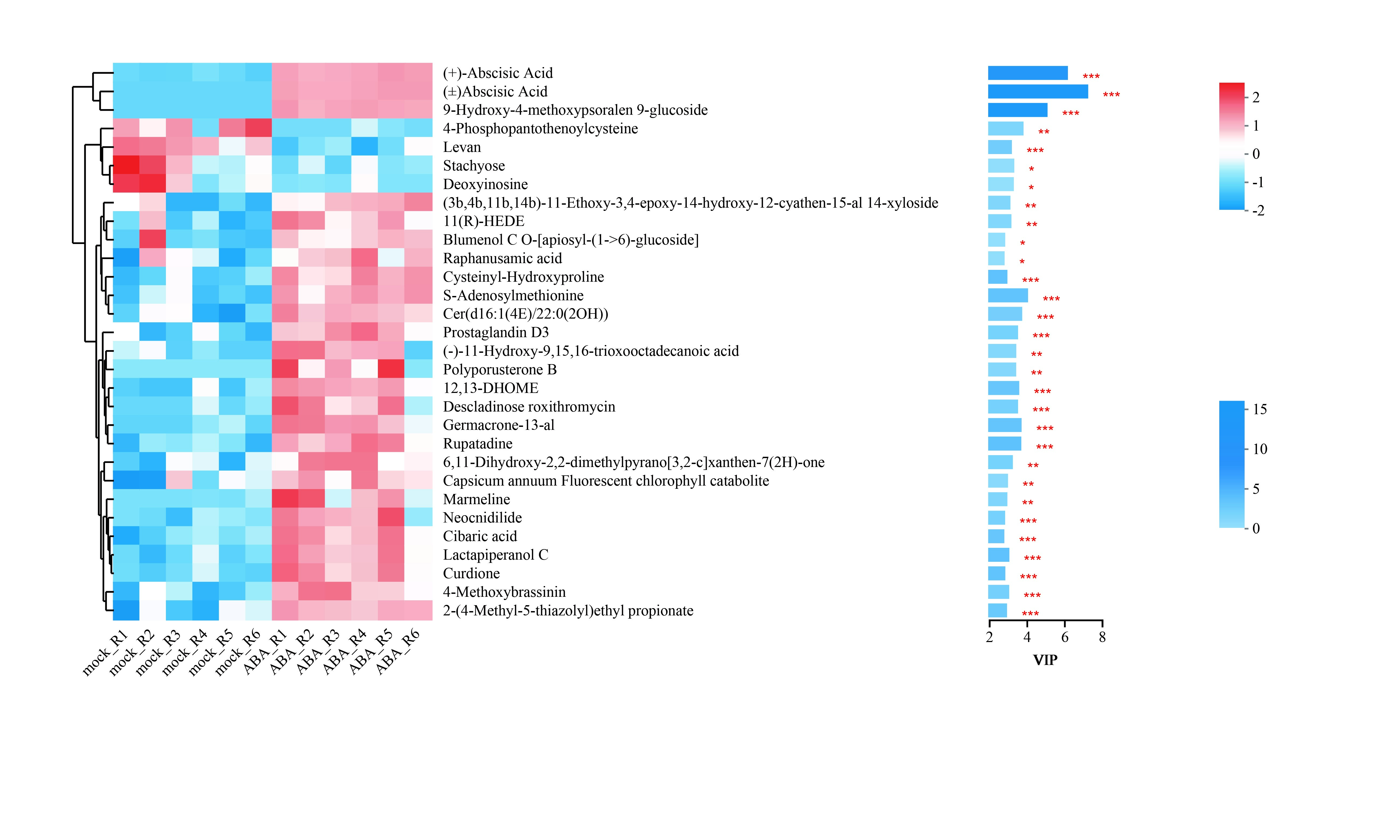


**Fig. S4** Expression profile and VIP of metabolites.

The expression profile and VIP of DEMs between mock and ABA-treated samples.


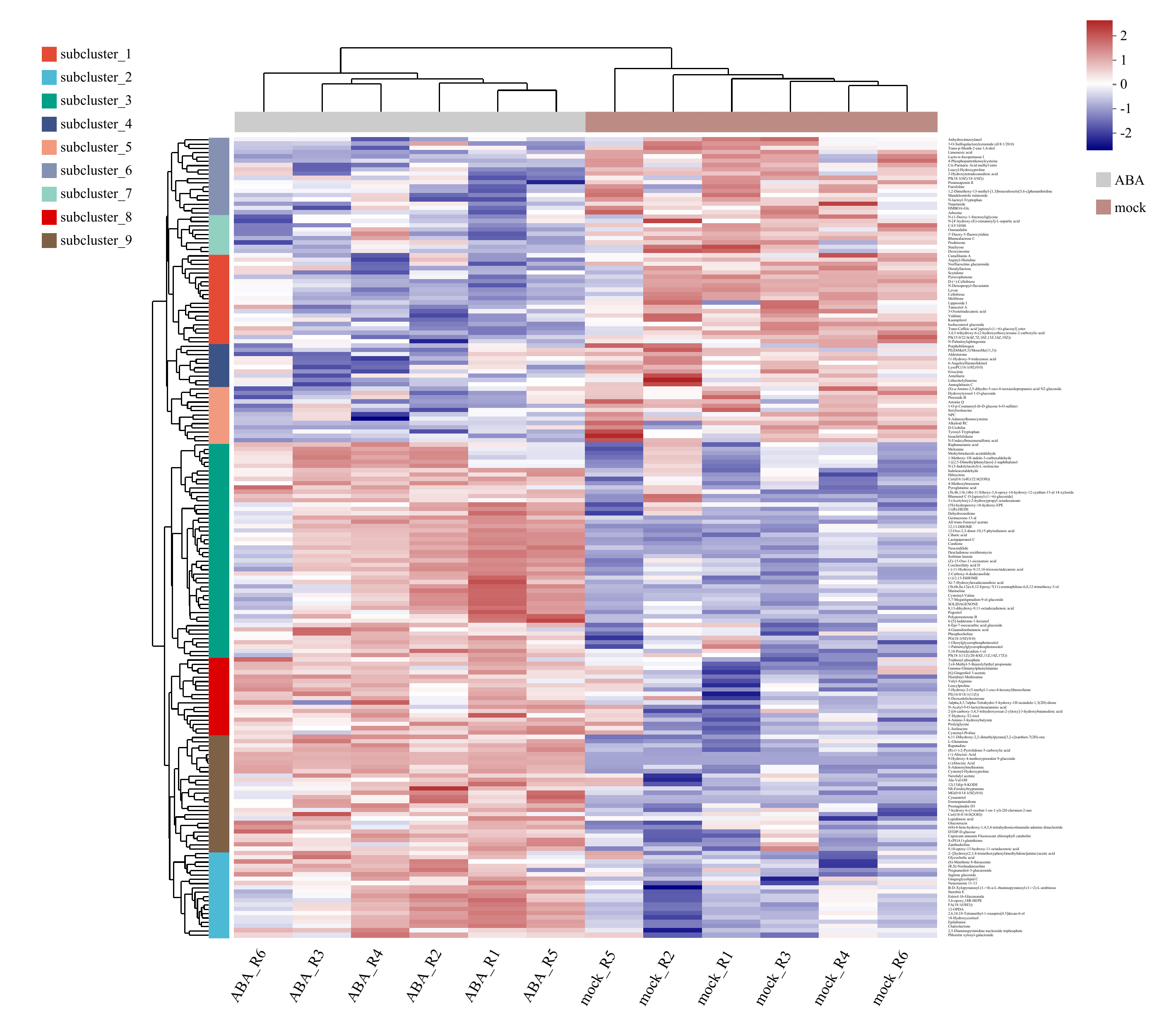


**Fig. S5** Heatmap with hierarchical clustering analysis of proportional content.

The heatmap with hierarchical clustering analysis of proportional content based on the expression level of all DEMs between mock and ABA-treated samples.
